# MassARRAY and SABER Analyses of SNPs in Embryo DNA Reveal the Abscission of Self-Fertilised Progeny during Fruit Development of Macadamia (*Macadamia integrifolia* Maiden & Betche)

**DOI:** 10.3390/ijms25126419

**Published:** 2024-06-11

**Authors:** Anushika L. De Silva, Wiebke Kämper, Steven M. Ogbourne, Joel Nichols, Jack W. L. Royle, Trent Peters, David Hawkes, Shahla Hosseini Bai, Helen M. Wallace, Stephen J. Trueman

**Affiliations:** 1 Centre for Planetary Health and Food Security, School of Environment and Science, Griffith University, Nathan, QLD 4111, Australia; anushika.desilva@griffithuni.edu.au (A.L.D.S.); j.nichols2@griffith.edu.au (J.N.); s.hosseini-bai@griffith.edu.au (S.H.B.); 2 Functional Agrobiodiversity and Agroecology, Department of Crop Sciences, University of Göttingen, 37077 Göttingen, Germany; wiebke.kaemper@uni-goettingen.de; 3 Centre for Bioinnovation, University of the Sunshine Coast, Maroochydore DC, QLD 4558, Australia; sogbourn@usc.edu.au; 4 School of Science, Technology & Engineering, University of the Sunshine Coast, Maroochydore DC, QLD 4558, Australia; 5 Australian Genome Research Facility, Gehrmann Laboratories, University of Queensland, Brisbane, QLD 4072, Australia; jack.royle@agrf.org.au (J.W.L.R.); trent.peters@agrf.org.au (T.P.); david.hawkes@agrf.org.au (D.H.); 6 School of Biology and Environmental Science, Queensland University of Technology, GPO Box 2434, Brisbane, QLD 4001, Australia; helen.wallace@qut.edu.au

**Keywords:** abscission, kernel, *Macadamia integrifolia*, outcrossing, premature fruit drop, Proteaceae, selective abortion, self-incompatibility, self-sterility, xenia

## Abstract

Yield in many crops is affected by abscission during the early stages of fruitlet development. The reasons for fruitlet abscission are often unclear but they may include genetic factors because, in some crops, self-pollinated fruitlets are more likely to abscise than cross-pollinated fruitlets. Pollen parentage can also affect final fruit size and fruit quality. Here, we aimed to understand the effects of pollen parentage on fruitlet retention and nut quality in orchards of macadamia (*Macadamia integrifolia* Maiden & Betche). We identified the pollen parent of macadamia ‘cultivar ‘816’ embryos by analysing single nucleotide polymorphisms (SNPs) in their DNA using customised MassARRAY and Single Allele Base Extension Reaction (SABER) methods. This allowed us to determine the proportions of self-fertilised and cross-fertilised progeny during premature fruit drop at 6 weeks and 10 weeks after peak anthesis, as well as at nut maturity. We determined how pollen parentage affected nut-in-shell (NIS) mass, kernel mass, kernel recovery, and oil concentration. Macadamia trees retained cross-fertilised fruitlets rather than self-fertilised fruitlets. The percentage of progeny that were cross-fertilised increased from 6% at 6 weeks after peak anthesis to 97% at nut maturity, with each tree producing on average 22 self-fertilised nuts and 881 cross-fertilised nuts. Three of the four cross-pollen parents provided fruit with significantly higher NIS mass, kernel mass, or kernel recovery than the few remaining self-fertilised fruit. Fruit that were cross-fertilised by ‘842’, ‘A4’, or ‘A203’ had 16–29% higher NIS mass and 24–44% higher kernel mass than self-fertilised fruit. Nuts that were cross-fertilised by ‘A4’ or ‘A203’ also had 5% or 6% higher kernel recovery, worth approximately $US460–540 more per ton for growers than self-fertilised nuts. The highly selective abscission of self-fertilised fruitlets and the lower nut quality of self-fertilised fruit highlight the critical importance of cross-pollination for macadamia productivity.

## 1. Introduction

The world’s population is expected to reach 9.7 billion by 2050 and a rapid increase in crop production is required to satisfy the rising global demand for food [1,2,3]. However, low and inconsistent crop yields create food production gaps in many agricultural systems [4]. Yield is limited in many fruit and nut crops by the premature abscission of fruitlets or seeds before maturity [5,6]. The vast majority of fruitlets that are formed 2–3 weeks after flowering in many tree crops subsequently abscise before maturity [7,8,9,10]. As a result, often less than 5% of flowers produce harvestable fruit [10,11,12,13,14].

The abscission of fruitlets can be exacerbated or caused by environmental, physiological, or genetic factors. Environmental and physiological factors such as high temperature, soil water deficit, and limited availability of carbohydrate and mineral resources may cause premature fruit drop [7,15,16,17,18,19,20]. Reducing the efflux of carbohydrate assimilates by girdling main branches can significantly decrease the number of fruitlets that abscise after the initial fruit set [21,22,23]. The application of mineral nutrients such as boron, potassium, or zinc can also decrease fruitlet abscission [6,16,24]. Genetic factors might also cause premature fruit abscission. For example, many plants abscise more selfed fruitlets than outcrossed fruitlets during the period of premature fruit drop as a result of inbreeding depression or late-acting self-incompatibility [25,26,27,28]. Inbreeding depression occurs when deleterious recessive alleles are expressed in the progeny of genetically related parents, causing reduced fitness [29,30,31,32,33]. Inbreeding depression can be expressed at any stage of the plant life cycle and it may manifest as the reduced vigour of selfed progeny, reducing their ability to compete against outcrossed progeny for the maternal resources required for fruit development [30]. In contrast, post-zygotic self-incompatibility is a genetically based self-recognition system that results in the complete failure of embryo development following self-fertilisation [33,34,35]. Fruitlet abscission due to post-zygotic self-incompatibility generally occurs at a uniform stage across the individuals of a population, following the abortion of a high proportion of the self-fertilised embryos [33,36,37,38].

Macadamia (*M. integrifolia* Maiden & Betche, *M. tetraphylla* L.A.S.Johnson and hybrids) is a subtropical crop that contributes over 62,000 tons of kernels annually to world tree-nut production [39]. Macadamia trees produce up to 3500 racemes each year [14,18,40], with each raceme bearing 100–300 bisexual flowers [9,14,41]. However, fewer than 2% of macadamia flowers develop into mature fruit [9,13,14,42]. Flower and premature fruit abscission in macadamia are most intense during three phases: (1) the abscission of flowers and fruitlets from anthesis to 2 weeks after peak anthesis; (2) the abscission of fruitlets from 5 to 7 weeks after peak anthesis; and (3) the abscission of fruitlets around 10 weeks after peak anthesis [9]. The first phase largely includes unfertilised flowers [41], whereas many fruitlets abscising around 5 weeks after peak anthesis appear to be fertilised [43]. Macadamia flowers are partially self-incompatible, with many self-pollen tubes arrested in the upper style before they can reach the lower style and ovary [42,44] and initial fruit set being higher after hand cross-pollination than hand self-pollination [45,46]. Recent evidence shows that almost all mature fruit of many macadamia cultivars are cross-fertilised [14,47,48,49,50,51]. However, it is unclear whether the initial fruit set and premature fruit drop in macadamia orchards comprise a mixture of self-fertilised and cross-fertilised fruitlets or, alternatively, if they comprise almost only cross-fertilised fruitlets. If the initial set includes a substantial proportion of self-fertilised fruitlets, then it is also unclear whether their subsequent abscission is due to inbreeding depression or post-zygotic incompatibility. An understanding of macadamia fruitlet paternity would help to determine whether premature fruit drop is driven more by genetic than environmental or physiological factors.

Pollen parentage may also strongly influence final fruit size and fruit quality [14,17,48,51,52,53,54,55,56]. The direct effect of the pollen genotype on the growth and morphology of the developing endosperm, embryo, or maternal tissue that results in differences in the characteristics of the fruit is termed ‘xenia’ [57]. Xenic effects can include differences in fruit or seed size, shape, colour, chemical composition, and the time taken to reach maturity [17,56,57,58]. Fruit growth rate, the timing of shell hardening, and the mature nut size of macadamia have been found to differ between two cross-pollen parents [17,56]. Recently, it has been shown that macadamia nut-in-shell (NIS) mass, kernel mass, and kernel recovery (i.e., the percentage of nut mass that comprises kernel) are also often higher in cross-fertilised fruit than self-fertilised fruit [14,48,51].

The objective of this study was to determine the effects of pollen parentage on the fruitlet retention, fruitlet development, and nut quality of macadamia. Specifically, we aimed to determine how the proportions of self-fertilised and cross-fertilised progeny change during fruit development. We used our existing MassARRAY method to determine the pollen parentage of macadamia fruitlets and mature fruit by analysing DNA sequences containing uniquely homozygous single nucleotide polymorphisms (SNPs) that we identified from each macadamia cultivar in a previous study [14]. We also developed a customised Single Allele Base Extension Reaction (SABER) technique, in combination with the MassARRAY platform, to confirm the pollen parent of young macadamia fruitlets. SABER was developed originally to discriminate SNPs in human foetal DNA among the much larger background of human maternal DNA in a maternal plasma sample [59]. Here, we used SABER to identify SNPs in plant embryo DNA among a background of endosperm DNA and potentially contaminating maternal-plant DNA in a fruitlet sample. Furthermore, we aimed to evaluate how pollen parentage affects fruitlet development and nut quality, including fruitlet mass, mineral nutrient levels in fruitlets and kernels, NIS mass, kernel mass, kernel recovery and kernel oil concentration. The results of this study will help to understand the driving factors behind premature fruit abscission in macadamia and the importance of pollen sources in determining nut quality.

## 2. Results

### 2.1. Fruitlet Retention

**Figure 1:**
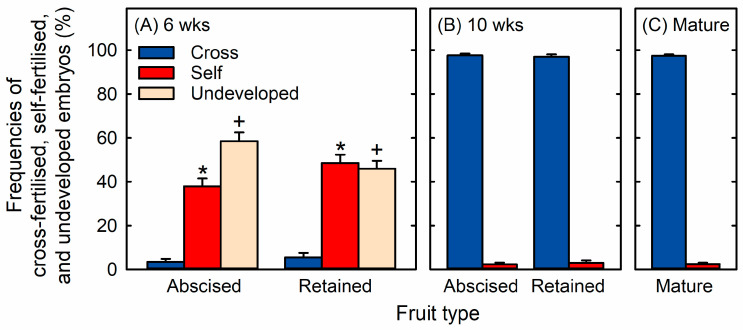
Frequencies of cross-fertilised, self-fertilised and undeveloped progeny of macadamia cv. ‘816’ trees among the (**A**) abscised fruitlets and retained fruitlets at 6 weeks after peak anthesis, (**B**) abscised fruitlets and retained fruitlets at 10 weeks after peak anthesis, and (**C**) mature fruit. Significant differences between means (+SE) for abscised fruitlets and retained fruitlets within a time point are denoted by an asterisk (*) or a plus (+) symbol (GLM, *p* < 0.05, n = 30 trees).

We aimed to determine whether the percentages of self- and cross-fertilised progeny changed during the fruit development of macadamia (*Macadamia integrifolia* Maiden & Betche) cultivar ‘816’. Most of the retained and the abscised fruitlets at 6 weeks after peak anthesis had an undeveloped embryo or were identified as self-fertilised by both MassARRAY and SABER (Figure 1A). Only 4 ± 1% of abscised fruitlets and 6 ± 2% of retained fruitlets at this stage were cross-fertilised (Figure 1A). SABER confirmed the results of the original MassARRAY analysis for every embryo collected at 6 weeks after peak anthesis.

All fruitlets at 10 weeks after peak anthesis had a developed embryo, and almost all of these were cross-fertilised (Figure 1B). By this stage, 98 ± 1% of abscised fruitlets and 97 ± 1% of retained fruitlets were cross-fertilised. Similarly, 97 ± 1% of the mature fruit were cross-fertilised (Figure 1C). Each tree of macadamia cultivar ‘816’ produced 22 ± 5 self-fertilised fruit and 881 ± 9 cross-fertilised fruit at maturity.

### 2.2. Biomass and Nutrient Accumulation in Fruitlets

Cultivars ‘842’, ‘741’, and ‘A4’ were the most common pollen parents of the retained fruitlets at 10 weeks after peak anthesis (Table S1). Very few fruitlets by this stage were self-fertilised (Figure 1B), and so there were insufficient self-fertilised fruitlets available to make comparisons between cross-fertilised and self-fertilised fruitlets. Fruitlets that were fertilised by ‘842’ were heavier than fruitlets fertilised by ‘741’, which were heavier than fruitlets fertilised by ‘A4’ (Table S1).

The fruitlets fertilised by ‘842’ had lower P, K, B, Mg, Na, and S concentrations than the fruitlets fertilised by either ‘741’ or ‘A4’ (Table S2). The concentrations of other nutrients did not differ significantly among the three cross-pollen parents (Table S2).

Fruitlets that were fertilised by ‘A4’ had lower total contents per fruitlet of N, P, K, B, Cu, and S than the fruitlets fertilised by ‘741’ or ‘842’ (Table S3). Furthermore, fruitlets fertilised by ‘A4’ had lower total Fe, Mg, Mn, and Zn contents per fruitlet than the fruitlets fertilised by ‘842’ (Table S3). Total aluminium and Na contents per fruitlet at 10 weeks after peak anthesis did not differ significantly among the three cross-pollen parents (Table S3).

### 2.3. Nut Quality

**Figure 2:**
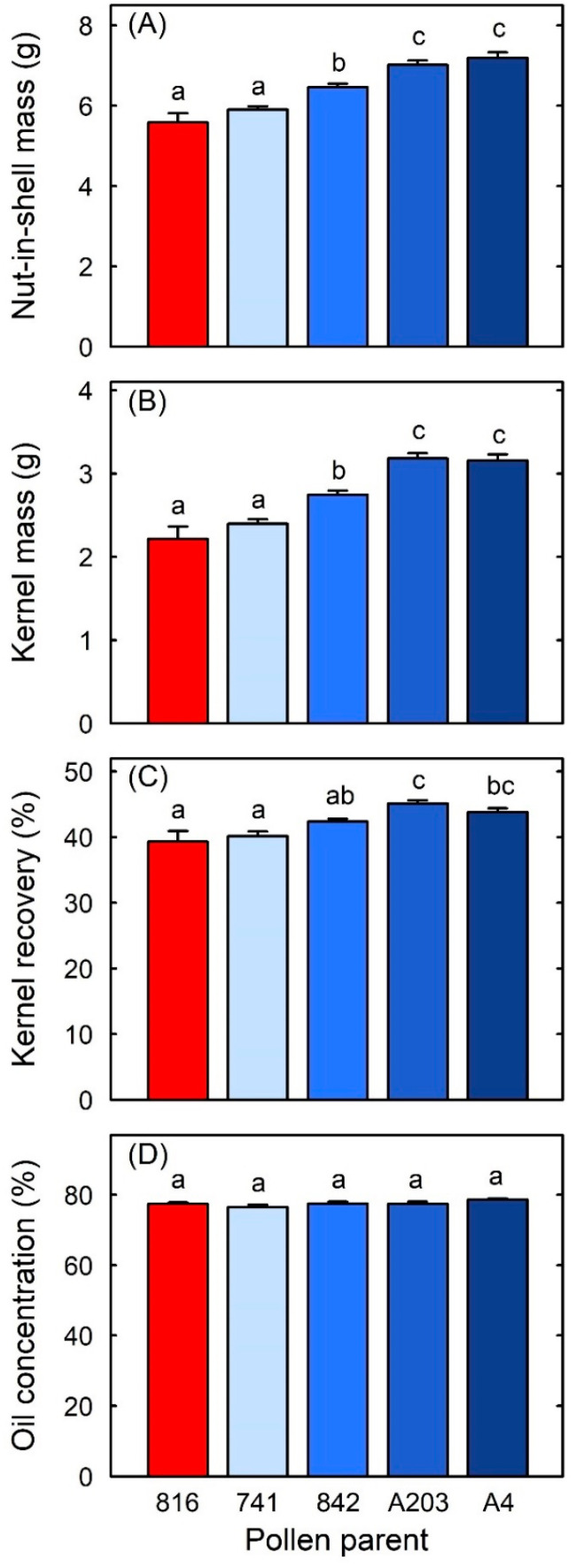
(**A**) Nut-in-shell (NIS) mass, (**B**) kernel mass, (**C**) kernel recovery and (**D**) kernel oil concentration of macadamia cultivar ‘816’ fruit arising from different pollen parents. Means (+SE) with different letters within a nut-quality parameter are significantly different (GLM, *p* < 0.05, n = 14 nuts for self-pollination, i.e., ‘816’, n = 73–124 nuts for other pollen parents).

Cross-fertilisation often produced mature fruit with higher NIS mass, kernel mass, and kernel recovery than self-fertilised fruit. Fruit that were fertilised by ‘842’, ‘A203’, or ‘A4’ had 16–29% higher NIS mass and 24–44% higher kernel mass than self-fertilised fruit (Figure 2A,B). Fruit fertilised by ‘A203’ or ‘A4’ also had 6% or 5% higher kernel recovery (in absolute terms), respectively, than self-fertilised fruit (Figure 2C). However, NIS mass, kernel mass, and kernel recovery did not differ significantly between fruit fertilised by ‘741’ and self-fertilised fruit (Figure 2A–C).

In addition, NIS mass, kernel mass, and kernel recovery differed among some cross-pollen parents. Fruit that were fertilised by either ‘A4’ or ‘A203’ had 9–22% higher NIS mass and 15–33% higher kernel mass than fruit fertilised by either ‘741’ or ‘842’ (Figure 2A,B). Fruit fertilised by ‘A203’ had 3–5% higher kernel recovery than fruit fertilised by ‘741’ or ‘842’ (Figure 2C). The kernel oil concentration did not differ significantly among pollen parents (Figure 2D). 

Cross-fertilised fruit had consistently higher kernel B concentrations and lower kernel Al concentrations than self-fertilised fruit (Table S4). Fruit that were fertilised by ‘A203’ had lower kernel N concentration than self-fertilised fruit or fruit fertilised by ‘842’ (Table S4). However, fruit fertilised by ‘741’ or ‘A203’ had higher kernel K concentration than self-fertilised fruit (Table S4). The concentrations of most other kernel nutrients did not differ significantly among pollen parents (Table S4). 

Fruit fertilised by ‘A4’, ‘A203’, or ‘842’ had higher total contents of either N, P, K, B, Fe, Mg, Mn, or S in the kernel than self-fertilised fruit (Table S5). Self-fertilised fruit had higher total kernel Al content than fruit fertilised by ‘741’ (Table S5). The total contents of Ca and Cu per kernel did not differ significantly among the pollen parents (Table S5).

## 3. Discussion

We developed a customised SABER technique in combination with the MassARRAY platform to identify SNPs in plant embryo DNA that reveal the pollen parent of young fruitlets. As a result, our study is the first to demonstrate that macadamia selectively abscises self-fertilised fruitlets and retains cross-fertilised fruitlets during premature fruit drop. Most of the fruitlets with developing embryos at 6 weeks after peak anthesis were self-fertilised but the mature fruit resulted almost entirely from cross-fertilisation. The study also demonstrated that self-fertilised fruit often have lower nut quality than cross-fertilised fruit and that nut quality differs among cross-pollen parents.

### 3.1. Fruitlet Retention

The MassARRAY and SABER analysis of SNPs in embryo DNA clearly revealed that macadamia selectively abscised self-fertilised fruitlets and retained cross-fertilised fruitlets between 6 and 10 weeks after anthesis, i.e., during the period of premature fruit drop. Fruit set at 6 weeks after anthesis comprised a much higher percentage of self-fertilised fruitlets than cross-fertilised fruitlets, with many other fruitlets having undeveloped embryos. All of the remaining fruitlets at 10 weeks after peak anthesis had a developed embryo, and almost all of these were cross-fertilised. Our study is the first to demonstrate that the initial fruit set of macadamia in a commercial orchard comprises mostly self-fertilised fruitlets, suggesting very strongly that most flowers receive self-pollen rather than cross-pollen. However, almost all of the self-fertilised fruitlets abscise by 10 weeks after peak anthesis. Selective abscission of self-fertilised fruitlets has been demonstrated in other tree crops such as avocado, lychee, and mango [25,26,27,60]. However, unlike macadamia, these crops retain a high percentage of selfed progeny among their mature fruit [11,25,27,60,61,62,63,64].

Selective abscission of self-fertilised macadamia fruitlets could be due to inbreeding depression or late-acting self-incompatibility. Inbreeding depression can involve the accumulation of lethal recessive alleles, resulting in seed abortion that can be very difficult to distinguish from that caused by late-acting self-incompatibility [34,65,66]. Inbreeding depression can also be caused by an accumulation of non-lethal deleterious alleles that cause slower growth of the self-fertilised embryos, reducing their capacity to compete with outcrossed embryos for maternal resources throughout the period of fruit development [30,34,37]. The proportion of fruitlets that were self-fertilised was very low at 10 weeks after peak anthesis, so we could not determine whether the selfed progeny were smaller or had lower mineral nutrient levels than the outcrossed progeny. However, we observed that the smaller fruitlets cross-fertilised by ‘A4’, which had lower total contents of all nutrients except Al and Na, competed effectively for biomass and mineral nutrient accumulation against the larger fruitlets cross-fertilised by ‘741’ or ‘842’. Cultivar ‘A4’ reaches anthesis later in the flowering period than ‘741’ and ‘842’ [67,68] and so the fruitlets cross-fertilised by ‘A4’ were probably younger than the fruitlets cross-fertilised by ‘741’ or ‘842’. Nonetheless, mature nuts that were cross-fertilised by ‘A4’ were heavily represented among the final harvest and they were ultimately larger than the mature nuts cross-fertilised by ‘741’ or ‘842’. This suggests that small fruitlet size, per se, was not the sole driving factor behind premature fruit abscission, at least among the outcrossed fruitlets.

Late-acting self-incompatibility would likely lead to a high proportion of the self-fertilised progeny abscising within a concentrated period [33,34,36,37,38,69]. Strongly selective abscission was evident among the macadamia fruitlets around 6 weeks after peak anthesis, so that the percentage of progeny on the tree that were cross-fertilised increased from only 6% at 6 weeks after peak anthesis to 97% at 10 weeks after peak anthesis. Most macadamia cultivars are partially self-incompatible in the pre-zygotic stage, with self-pollen tubes often arrested in the style before they can reach the ovary [44,45]. Our results demonstrate that most of the initially set fruitlets were self-fertilised despite the pre-zygotic mechanisms that select against self-fertilisation. Failed embryo development after self-fertilisation is another form of self-incompatibility but expressed in the post-zygotic stage [33]. The ovules of abscised, fertilised fruitlets of macadamia cv. ‘246’ appear similar microscopically at 5 weeks after anthesis to those of actively growing fruitlets, although some have irregularly shaped embryos with crushed cells [43]. These observations for cv. ‘246’, in combination with the observed heavy abscission of both the undeveloped and the selfed cv. ‘816’ fruitlets in a concentrated period, suggest that at least some abscission during the early stages of premature fruit drop in macadamia may be the result of late-acting self-incompatibility.

Almost all of the abscised fruitlets at 10 weeks after peak anthesis were cross-fertilised, which suggests that environmental or physiological factors may be the major cause of fruitlet abscission during the later stages of premature fruit drop. The number of remaining fruitlets may have been above the resource-provisioning capacity of the tree [15]. Fruitlet abscission at this stage could be an adaptive mechanism by which crop load is adjusted to match the available maternal resources, prior to the major period of fruit-biomass accumulation [9,15,70]. Tree management practices such as branch girdling and canopy pruning affect the levels of assimilates in macadamia trees and alter the capacity of trees to retain developing fruitlets [18,71,72]. Girdling small branches that carry high numbers of leaves can increase the accumulation of carbohydrates and reduce the abscission of macadamia fruitlets [9,17]. Girdling the larger main branches can also help to maintain high carbohydrate levels in fruiting branches and alleviate fruitlet abscission [72,73]. The early stages of macadamia fruit development are more sensitive than the later stages to fluctuations in tree carbohydrate levels [72]. Pruning the canopy during later stages rather than early stages of fruit development can avoid reductions in carbohydrate levels during early fruit development [18]. Therefore, strategic timing of canopy management can be used to maintain high carbohydrate levels within the tree during spring and increase fruitlet retention during the later stages of premature fruit drop.

### 3.2. Nut Quality

MassARRAY analysis of SNPs in kernel DNA also revealed that pollen parentage greatly affected the quality of mature macadamia nuts. Cross-fertilised fruit often had higher nut-in-shell (NIS) mass, kernel mass, and kernel recovery than self-fertilised fruit, depending on the cross-pollen parent. Differences in fruit quality between pollen parents can be attributed directly to seed paternity in single-seeded fruit, such as almond, avocado, hazelnut, and macadamia, without the confounding effect of different numbers of seeds per fruit [14,27,52,54,74]. The kernel is the embryo of the macadamia fruit [75,76] and so this tissue has ~50% maternal and ~50% paternal genetic contribution. Therefore, it was not surprising that cross-fertilisation affected both the kernel mass and the percentage of NIS mass that was composed of kernel mass, i.e., kernel recovery. Nut quality also differed significantly between some cross-pollen parents, with ‘A4’ as the cross-pollen parent providing fruit with higher NIS mass, kernel mass, or kernel recovery than either ‘741’ or ‘842’ as the cross-pollen parent. Cultivar ‘A4’ trees generally produce fruit with higher NIS mass, kernel mass, and kernel recovery than trees of cultivars ‘741’ and ‘842’ [77]. Our results show that these characteristics are also imparted on the progeny when ‘A4’ is the father of the fruit.

These xenic effects on nut quality affect financial returns for macadamia growers and processors. Growers are paid a premium for nuts with higher kernel recovery [78,79]. Nuts from the self-fertilised fruit in our study had 39% kernel recovery, worth $US3570 per ton at farmgate prices of $US3000 per ton of nuts at 33% kernel recovery. Fruit that were cross-fertilised by ‘A4’ or ‘A203’ had 44% and 45% kernel recovery, making them worth $US460 and $US540 more per ton, respectively, than the self-fertilised fruit. They were also worth $US380 and $US460 more per ton, respectively, than the fruit cross-fertilised by ‘741’. Macadamia kernels are subsequently categorised by processors into a range of ‘styles’, with the styles that comprise large kernels and whole kernels—rather than small kernels and halves or pieces—having higher market value [80,81]. Fruit and nut orchards are often established with wide blocks of single cultivars to reduce the complexity of orchard management operations such as irrigation, fertiliser application, pest and disease control, and harvesting [63,82,83,84,85]. However, these wide-block orchard designs can reduce the opportunities for the production of cross-fertilised fruit, as pollen needs to be transported across many orchard rows from one cultivar to another cultivar to effect cross-pollination [14,48,63,85,86,87]. This can lead to reduced financial value if it causes significant proportions of the crop to be produced by self-pollination or by cross-pollen sources that do not provide optimal product quality [14,51,53,88,89,90]. Therefore, the strong xenic effects on macadamia nut quality and product value highlight that the strategic selection and placement of cultivars as both nut producers and cross-pollinisers could provide growers and processors with higher economic returns.

## 4. Materials and Methods

### 4.1. Study Site and Sampling Design

We conducted the study in a commercial orchard of macadamia (*M. integrifolia* Maiden & Betche, *M. tetraphylla* L.A.S.Johnson, and hybrids) at Alloway near Bundaberg, Queensland, Australia. The site (24°56′6″ S 152°21′16″ E) contained 13- and 16-year-old trees. We selected thirty trees in the middle row of a five-row-wide block of cultivar ‘816’ trees that were 13 years old, as described previously [47]. This block of ‘816’ trees was surrounded by five-row-wide blocks of cultivars ‘741’, ‘842’, ‘A203’, and ‘A4’. The experimental ‘816’ trees were divided into ten plots along the row, with each plot containing three experimental trees as well as non-experimental buffer trees. Each experimental tree within a plot was randomly allocated to one of three boron fertiliser treatments (0, 15 or 30 g B per tree) that were applied prior to flowering. These treatments had no significant effect on nut yield, nut quality, or nut paternity [47].

### 4.2. Sample Collection and Processing

**Figure 3:**
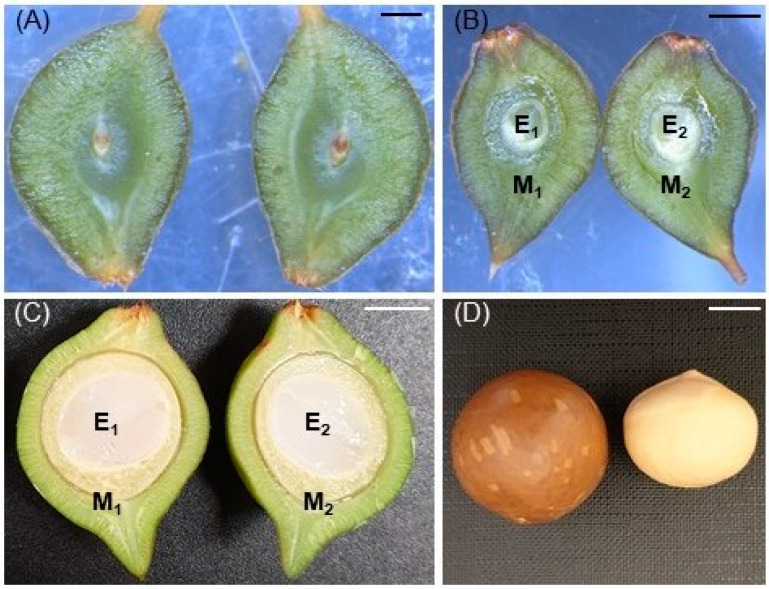
(**A**) Cross section of a macadamia fruitlet with an undeveloped embryo at 6 weeks after peak anthesis (scale bar = 0.7 mm), (**B**) cross section of a developing macadamia fruitlet at 6 weeks after peak anthesis (scale bar = 2.5 mm), (**C**) cross section of a developing macadamia fruitlet at 10 weeks after peak anthesis (scale bar = 4.5 mm), and (**D**) macadamia nut-in-shell and kernel at maturity (scale bar = 10 mm). E_1_ and E_2_ = syngamous tissue (embryo and endosperm) of the two halves of the fruitlet; M_1_ and M_2_ = developing shell and husk of the two halves of the fruitlet.

We sampled ten fruitlets within the canopy of each tree and ten freshly abscised fruitlets under each tree at both 6 and 10 weeks after peak anthesis. Fruitlets from the canopy were sampled at 1.0–1.5 m above the ground. One fruitlet was collected from the canopy margin and one fruitlet was collected halfway into the canopy, at each of the four cardinal directions on the tree, and two fruitlets were collected in the canopy close to the trunk. Similarly, one abscised fruitlet was collected from the orchard floor under the canopy margin, one abscised fruitlet was collected from the orchard floor halfway into the canopy at each of the four cardinal directions, and two abscised fruitlets were collected from the orchard floor close to the trunk. The samples were transferred to the laboratory immediately after collection and stored at −18 °C. Each fruitlet was later thawed and dissected longitudinally into two halves to expose the syngamous tissue comprising the embryo and possibly endosperm (hereafter ‘embryo’) (Figure 3A–C). The whole developing embryo of each fruitlet was used to determine the pollen parentage of each fruitlet collected at 6 weeks after peak anthesis (Figure 3B). A subsample of approximately 70 mg from the embryo of one-half of the fruitlet (E_2_) was used to determine the pollen parentage of fruitlet samples collected at 10 weeks after peak anthesis (Figure 3C). The other half of each of the fruitlets collected from the tree canopy (E_1_ + M_1_) was used to measure mineral nutrient concentrations (Figure 3C). All samples were weighed.

We also sampled 20 mature fruit from the final harvests of each tree to assess paternity, nut quality, and mineral nutrient concentrations. The random sampling method for fruit from the final harvests was described previously [47]. The fruit were dehusked and then the nuts (Figure 3D) were dried at 37 °C for 2 d, 45 °C for 2 d, and 57 °C for 2 d [91]. We recorded nut-in-shell (NIS) mass for each nut. Each nut was cracked manually using a TJ’s nutcracker (T.J.’s, Morayfield, Australia) and the mass of its kernel (Figure 3D) was also recorded. Kernel recovery was calculated as the percentage (*w*/*w*) of NIS that comprised kernel [47]. Each kernel was then dissected into three representative sub-samples of at least: (a) 70 mg for paternity analysis; (b) 300 mg for mineral nutrient analysis; and (c) 500 mg for the determination of oil concentration.

### 4.3. Paternity Analysis

Each embryo or kernel subsample was combined with disposable 0.1 mm and 2.3 mm diameter zirconia/silica beads (Daintree Scientific, St Helens, Australia) and frozen in liquid nitrogen before grinding into a powder using an MM2000 TissueLyser (Retsch, Haan, Germany) [54]. DNA extraction followed the glass-fibre DNA-extraction protocol for plants [92]. We analysed a subset of 438 uniquely homozygous single nucleotide polymorphisms (SNPs) that we identified previously from macadamia cultivars [14] to determine the pollen parentage of each fruitlet or mature fruit (Table 1). The pollen parentage of all samples was assigned using the Agena MassARRAY platform (Agena Bioscience, San Diego, CA, USA) [14]. The MassARRAY identifies SNPs that are both homozygous and unique to each macadamia cultivar (Table 1) and so can be used to definitively identify the pollen-parent cultivar of each embryo or kernel. A self-fertilised embryo or kernel results in all assays providing a homozygous signal, whereas a cross-fertilised embryo or kernel results in the assays providing uniquely identifying heterozygous signals that reveal the pollen parent [14]. 

A highly sensitive SABER technique [59] in combination with the Agena MassARRAY platform was also designed as a method to confirm the MassARRAY results for the paternity of the fruitlets collected at 6 weeks after peak anthesis. We customised the SABER method as a means to confirm paternity because the genetic contribution from the pollen parent may be much lower than 50% in young embryo samples if the dissected sample also contains endosperm and surrounding maternal tissue. In that case, the MassARRAY could, potentially, provide a false ‘self-fertilised’ result for cross-fertilised embryos if the paternal DNA contribution was not distinguished from a larger background of maternal DNA. PCR products in both the standard and SABER protocols are subjected to base extension. Standard protocols involve the extension of both the common maternal allele (i.e., the common macadamia allele) and the alternative allele (i.e., the private cultivar allele; Table 1) together. However, each SABER assay extends only the alternative allele that may be provided by each cross-pollen parent or, separately, only the common maternal allele [59]. The extension of only the alternative (non-maternal) allele in each SABER assay, therefore, allows robust identification of a potentially low-abundance paternal allele in a sample of embryo tissue (~50% paternal genetic contribution) that might also contain endosperm (~33% paternal contribution) and, potentially, maternal tissue (0% paternal contribution). 

Multiplex PCR reactions for SABER were grouped together according to the targeted extension base, and the extracted embryo DNA (2 µL; ~10 ng/µL) was amplified using the PCR protocol described previously [14]. Unincorporated dNTPs were deactivated using 0.5 U of shrimp alkaline phosphatase (37 °C for 40 min, 85 °C for 5 min). Primer extension was initiated by adding 1 U of iPLEX Pro, 3 pmol of the relevant acyclonucleotide (New England BioLabs, Ipswich, MA, USA), and extension primers. The reaction conditions were 95 °C for 30 s, 40 cycles of 95 °C for 5 s plus five inner cycles of 52 °C for 5 s and 80 °C for 5 s, and a final extension at 72 °C for 3 min. Cation exchange, SpectroCHIP loading, and MALDI-TOF MS were performed as described previously [14].

The numbers of mature self- and cross-fertilised fruit per tree were then calculated using Equations (1) and (2):

*N_S_* = *Y*/*M* × *P_S_* (1)

*N_C_* = *Y*/*M* × *P_C_* (2)

where *N_S_* was the number of self-fertilised fruit, *Y* was the NIS yield of the tree, *M* was the average NIS mass of the tree, *P_S_* was the proportion of self-fertilised fruit in the tree, *N_C_* was the number of cross-fertilised fruit, and *P_C_* was the proportion of cross-fertilised fruit in the tree.

### 4.4. Mineral Nutrient Analysis

We determined the nitrogen (N) concentration of fruitlet and kernel subsamples by combustion analysis [93,94] using a LECO 928 Macro Determinator (LECO, Saint Joseph, MI, USA). We determined aluminium (Al), boron (B), calcium (Ca), copper (Cu), iron (Fe), magnesium (Mg), manganese (Mn), phosphorus (P), potassium (K), sodium (Na), sulphur (S), and zinc (Zn) concentrations by inductively coupled plasma atomic emission spectroscopy after nitric and perchloric acid digestion [95,96]. The mineral nutrient contents of each fruitlet collected at 10 weeks after peak anthesis were calculated using Equations (3)–(5):

*C*(*_E_*_1+_*_M_*_1_) = *A* × *M*(*_E_*_1+_*_M_*_1_) (3)

where *C*_(_*_E_*_1+_*_M_*_1)_ was the mineral nutrient content of the subsample *E*_1_ + *M*_1_, *A* was the mineral nutrient concentration of the subsample *E*_1_ + *M*_1_, and *M*_(_*_E_*_1+_*_M_*_1)_ was the mass of the subsample *E*_1_ + *M*_1_;

*C*(*_E_*_2+_*_M_*_2_) = *A* × *M*(*_E_*_2+_*_M_*_2_) (4)

where *C*_(_*_E_*_2+_*_M_*_2)_ was the mineral nutrient content of the subsample *E*_2_ + *M*_2_, *A* was the mineral nutrient concentration of the subsample *E*_1_ + *M*_1_, and *M*_(_*_E_*_2+_*_M_*_2)_ was the mass of the subsample *E*_1_ + *M*_1_; and

*C_F_* = *C*(*_E_*_1+_*_M_*_1_) + *C*(*_E_*_2+_*_M_*_2_) (5)

where *C_F_* was the mineral nutrient content of the whole fruitlet. The mineral nutrient contents of each kernel were calculated by multiplying each mineral nutrient concentration by the kernel mass.

### 4.5. Determination of Oil Concentration

We measured the specific gravity of each kernel using a pan immersed in a 95% (*v*/*v*) ethanol solution [97,98]. The oil concentration of each kernel was calculated using Equations (6) and (7):

Oil concentration (%) = 284.7 − 212.57 × specific gravity(6)

where:

Specific gravity = (0.7995 × mass in air)/(mass in air − mass in 95% ethanol)(7)

### 4.6. Relationships between Paternity and Fruitlet or Nut Quality

The effects of paternity on nutrient accumulation in non-abscising fruitlets sampled at 10 weeks after peak anthesis and on the quality of mature nuts were assessed when sufficient paternal diversity was available for comparisons. We included in the analyses only those pollen parents that provided at least 14 fruitlets or nuts, as other pollen parents produced less than 10 fruitlets or nuts.

### 4.7. Statistical Analysis

We compared the proportions of undeveloped, self-fertilised, and cross-fertilised embryos between three time points, viz. 6 weeks after peak anthesis, 10 weeks after peak anthesis, and at maturity, for both retained fruit and abscised fruit, using generalised linear models (GLMs) (SPSS version 26, SPSS Science, Chicago, IL, USA). Fixed effects were time after peak anthesis, boron treatment, and plot. We compared differences in the number of self- and cross-fertilised fruit per tree at maturity using GLMs. Fixed effects were boron treatment and plot. We also tested the effects of pollen parentage on the fresh mass of fruitlet components, on the nutrient levels of fruitlets, and on the NIS mass, kernel mass, kernel recovery, kernel oil concentration, and kernel nutrient levels of mature nuts using GLMs. Each fruitlet or fruit was considered an individual replicate because its paternity was considered to be based on an individual pollination event. Fixed effects were pollen parent, boron treatment, and plot. We compared differences between means using a pairwise comparison procedure with sequential Šidák’s corrections when significant differences were detected. Differences between means were regarded as significant at *p* < 0.05. Means are reported with standard errors.

## 5. Conclusions

A customised MassARRAY and SABER technique for analysing SNPs in plant embryo DNA revealed that the initial fruit set of macadamia trees in a commercial orchard was comprised mostly of fruitlets with either an undeveloped embryo or a self-fertilised embryo rather than a cross-fertilised embryo. This indicated that very few flowers were cross-pollinated. Almost all of the undeveloped and self-fertilised fruitlets had abscised by 10 weeks after peak anthesis, leaving almost only cross-fertilised fruitlets on the tree. The results suggest that inbreeding depression or post-zygotic self-incompatibility are driving the abscission of selfed progeny during the early stages of premature fruit drop in macadamia. The few remaining self-fertilised fruit at maturity had lower nut-in-shell (NIS) mass, kernel mass, and kernel recovery than many of the cross-fertilised fruit, with this effect depending on the cross-pollen source. As a result, self-fertilised nuts were worth $US460–$US540 less per ton than many of the cross-fertilised nuts. Growers could maximise the productivity of macadamia orchards by increasing the opportunities for optimal cross-pollination through closely interplanting different cultivars, strategically selecting polliniser cultivars, and widely distributing bee hives throughout the orchard.

## Figures and Tables

**Table 1:** Eight of the DNA sequences used for identifying pollen parents through MassARRAY and SABER analysis of unique homozygous SNPs from each macadamia cultivar, as described previously [14].

Cultivar	Common Macadamia Allele	Private Cultivar Allele	DNA Sequence with SNP	816	741	842	A203	A4
816	T	**C**	GGTCGTTACGCNGGCAGAAAAGCNGTAATAGTTNAGATCCTTCGACGATGGAACNTCGAGACCGTCCTTACGGCCATTGCTTGGT[**T/C**]GNCAGGAATCNGCCAAATACCCCAAGAAGGTNATTCGCAAGGATTCAGCNAAG	**CC**	**TT**	**TT**	**TT**	**TT**
741	G	**T**	TGCAGATTNCAATGTAAGAGGTACTAACCCANAACGAAGACAACTN[**G/T**]TCACTTTCTTTCCATTTGGCAAGCTNCCACTTGCTTTCTGCATCCANAANGAACACGTATTATATGACAAAAACAAGACTAAGTTNTCCAACAAAGA	**GG**	**TT**	**GG**	**GG**	**GG**
741	T	**A**	TGCAGGAATCTATCCTGCTATGGCAAAGAAAATGCGAGGATGCTCTGTATTCCCTNTCTCATTCTTGGTGCTCGTCNGGCCTGTAAGGCGTTTGGCATCATTAGN[**T/A**]ATGGCGAGAATTATATTAAAGGGTGATGGCATTTCG	**TT**	**AA**	**TT**	**TT**	**TT**
741	C	**T**	TGCAGGAATCTATCCTGCTATGGCAAAGAAAATGCGAGGATGCTCTGTATTCCCTNTCTCATTCTTGGTGCTCGTCNGGCCTGTAAGGCGTTTGGCATCATTAG[**C/T**]NATGGCGAGAATTATATNTAAAGGGTGATGGCATTTCG	**CC**	**TT**	**CC**	**CC**	**CC**
842	A	**G**	TTATTAATGCTCCTATCTCCTCCCTGTNTCCTGTTCCCTCAAGATTCCGCAGTTGGGCTTCTN[**A/G**]GCTTAGATATGTTCTCTAGCCATCAATTTTTTAATTTGNGACTATCTCCTTCTTTCTGGNGNAAGAATTTGGACC	**AA**	**AA**	**GG**	**AA**	**AA**
A203	T	**C**	TGCAGAACCAAGAGAGCACCATNCGCAACAACTCTTAGAACACNTGCACAATGACTAAGANTGCAGGATTATTTACTAN[**T/C**]TGCTTGCCACTTTTGAAACTAAGAACNAATATCTTCCATTCCTGANAGAAACAACTCCAAACAG	**TT**	**TT**	**TT**	**CC**	**TT**
A4	G	**A**	TGCAGCACAGTGGCATTGTANTGCAATTTCGTTGGAGCAGGNGGGGAGAGGGTAAAGGACTTCAAAAGATGTTTCN[**G/A**]AATATCTTGCAGAAGGCTTAGTCCTACTNTNTTGAGACCATTAATTAGTTGTATGAATATGAACTACA	**GG**	**GG**	**GG**	**GG**	**AA**
A4	G	**T**	TGCAGTTTCAGCAATCGACTCTGCTTCGATACGTGTGAGCNTCCTCACTGACAATGTCAGCAGCTTCACCAA[**G/T**]NGGCAAGAGCAATAGCAGCTTGAGCTTTTGTNGACTGGCTTATCTGGCTGAANAAAGTCTCGTGTAACCTG	**GG**	**GG**	**GG**	**GG**	**TT**

## Data Availability

The data will be made available upon request with permission from Hort Innovation.

## Supplementary Materials

The following supporting information can be downloaded at: https://www.mdpi.com/article/10.3390/ijms25126419/s1.
