# Supplementary material for: MassARRAY and SABER Analyses of SNPs in Embryo DNA Reveal the Abscission of Self-Fertilised Progeny during Fruit Development of Macadamia (Macadamia integrifolia Maiden & Betche)
Source: Int J Mol Sci. 2024 Jun 11;25(12):6419. doi: 10.3390/ijms25126419 (PMC11204000; doi:10.3390/ijms25126419)
Supplement: Supplementary file 1 [file ijms-25-06419-s001.zip › ijms-3031881-supplementary.pdf]

Supplementary File

MassARRAY and SABER Analyses of SNPs in Embryo DNA Reveal the Abscission of Self-Fertilised Progeny during Fruit Development of Macadamia (*Macadamia integrifolia* Maiden & Betche)

Anushika L. De Silva [1], Wiebke Kämper [2], Steven M. Ogbourne [3,4], Joel Nichols [1], Jack W.L. Royle [5], Trent Peters [5], David Hawkes [5], Shahla Hosseini Bai [1], Helen M. Wallace [6], Stephen J. Trueman [1,*]

[1] Centre for Planetary Health and Food Security, School of Environment and Science, Griffith University, Nathan, QLD 4111, Australia; anushika.desilva@griffithuni.edu.au (A.L.D.S.); j.nichols2@griffith.edu.au (J.N.); s.hosseini-bai@griffith.edu.au (S.H.B.)

[2] Functional Agrobiodiversity and Agroecology, Department of Crop Sciences, University of Göttingen, 37077, Göttingen, Germany; wiebke.kaemper@uni-goettingen.de (W.K.)

[3] Centre for Bioinnovation, University of the Sunshine Coast, Maroochydore DC, QLD 4558, Australia; sogbourn@usc.edu.au (S.M.O.)

[4] School of Science, Technology & Engineering, University of the Sunshine Coast, Maroochydore DC, QLD 4558, Australia

[5] Australian Genome Research Facility, Gehrmann Laboratories, University of Queensland, Brisbane, QLD 4072, Australia; jack.royle@agrf.org.au (J.W.L.R.); trent.peters@agrf.org.au (T.P.); david.hawkes@agrf.org.au (D.H.)

[6] School of Biology and Environmental Science, Queensland University of Technology, GPO Box 2434, Brisbane, QLD 4001, Australia; helen.wallace@qut.edu.au (H.M.W.)

* Correspondence: s.trueman@griffith.edu.au

Table S1. Fresh mass (g) of embryo, maternal tissue and total fruitlet at 10 weeks after peak anthesis of retained macadamia cultivar '816' fruitlets arising from different pollen parents

Component	Maternal parent × pollen parent		
	'816' × '741'	'816' × '842'	'816' × 'A4'
Embryo	**0.71 ± 0.14 b**	**1.13 ± 0.14 a**	**0.36 ± 0.03 c**
Maternal tissue	**5.34 ± 0.43 b**	**6.83 ± 0.49 a**	**4.47 ± 0.17 c**
Total fruitlet	**6.05 ± 0.55 b**	**7.96 ± 0.63 a**	**4.83 ± 0.20 c**

Means ± SE within a row with bold font and different letters are significantly different (GLM, $p < 0.05$, n = 27–111).

Table S2. Mineral nutrient concentrations (mg / 100 g) at 10 weeks after peak anthesis of retained macadamia cultivar '816' fruitlets arising from different pollen parents

Nutrient	Maternal parent × pollen parent		
	'816' × '741'	'816' × '842'	'816' × 'A4'
Nitrogen (N)	496 ± 10 a	481 ± 11 a	496 ± 6 a
Phosphorus (P)	**43.8 ± 0.9 a**	**39.1 ± 1.2 b**	**44.7 ± 0.5 a**
Potassium (K)	**250 ± 6 a**	**206 ± 6 b**	**256 ± 4 a**
Aluminium (Al)	0.148 ± 0.019 a	0.117 ± 0.018 a	0.164 ± 0.017 a
Boron (B)	**0.613 ± 0.081 a**	**0.517 ± 0.037 b**	**0.537 ± 0.024 ab**
Calcium (Ca)	56.5 ± 4.0 a	50.8 ± 3.9 a	63.4 ± 3.2 a
Copper (Cu)	0.198 ± 0.015 a	0.166 ± 0.009 a	0.186 ± 0.008 a
Iron (Fe)	1.24 ± 0.09 a	1.11 ± 0.11 a	1.41 ± 0.07 a
Magnesium (Mg)	**35.3 ± 1.0 ab**	**32.0 ± 1.1 a**	**36.8 ± 0.5 b**
Manganese (Mn)	0.695 ± 0.055 a	0.637 ± 0.070 a	0.784 ± 0.047 a
Sodium (Na)	**6.39 ± 0.81 ab**	**4.26 ± 0.39 a**	**7.22 ± 0.55 b**
Sulphur (S)	**42.1 ± 8.0 ab**	**38.8 ± 1.2 a**	**44.3 ± 0.7 b**
Zinc (Zn)	0.315 ± 0.013 a	0.294 ± 0.013 a	0.314 ± 0.007 a

Means ± SE within a row with bold font and different letters are significantly different (GLM, $p < 0.05$, n = 27–111).

Table S3. Total mineral nutrient contents per fruitlet (mg / fruitlet) at 10 weeks after peak anthesis of retained macadamia cultivar '816' fruitlets arising from different pollen parents

Nutrient	Maternal parent × pollen parent		
	'816' × '741'	'816' × '842'	'816' × 'A4'
Nitrogen (N)	**29.1 ± 2.4 a**	**37.6 ± 2.9 b**	**23.7 ± 0.9 c**
Phosphorus (P)	**2.55 ± 0.20 a**	**3.00 ± 0.21 a**	**2.12 ± 0.08 b**
Potassium (K)	**14.3 ± 1.1 a**	**15.7 ± 1.0 a**	**11.9 ± 0.4 b**
Aluminium (Al)	0.009 ± 0.002 a	0.009 ± 0.002 a	0.008 ± 0.001 a
Boron (B)	**0.032 ± 0.004 a**	**0.039 ± 0.003 a**	**0.025 ± 0.001 b**
Calcium (Ca)	**3.20 ± 0.29 a**	**3.87 ± 0.37 b**	**2.88 ± 0.16 a**
Copper (Cu)	**0.012 ± 0.002 a**	**0.013 ± 0.001 a**	**0.009 ± 0.001 b**
Iron (Fe)	**0.073 ± 0.009 ab**	**0.087 ± 0.013 a**	**0.067 ± 0.004 b**
Magnesium (Mg)	**2.04 ± 0.16 ab**	**2.42 ± 0.15 a**	**1.73 ± 0.06 b**
Manganese (Mn)	**0.043 ± 0.006 ab**	**0.048 ± 0.005 a**	**0.035 ± 0.002 b**
Sodium (Na)	0.335 ± 0.039 a	0.320 ± 0.032 a	0.313 ± 0.021 a
Sulphur (S)	**2.47 ± 0.20 a**	**2.97 ± 0.19 b**	**2.07 ± 0.07 c**
Zinc (Zn)	**0.019 ± 0.002 ab**	**0.023 ± 0.002 a**	**0.016 ± 0.001 b**

Means ± SE within a row with bold font and different letters are significantly different (GLM, $p < 0.05$, n = 27–111).

Table S4. Mineral nutrient concentrations (mg / 100 g) in mature kernels of macadamia cultivar '816' arising from different pollen parents

Nutrient	Maternal parent × pollen parent				
	'816' × '816'	'816' × '741'	'816' × '842'	'816' × 'A203'	'816' × 'A4'
Nitrogen (N)	**1444 ± 46 a**	**1310 ± 28 ab**	**1386 ± 22 a**	**1273 ± 24 b**	**1320 ± 33 ab**
Phosphorus (P)	210 ± 18 a	198 ± 13 a	195 ± 8 a	208 ± 9 a	204 ± 11 a
Potassium (K)	**292 ± 34 a**	**379 ± 25 bc**	**362 ± 15 ab**	**402 ± 15 c**	**364 ± 15 abc**
Aluminium (Al)	**0.252 ± 0.048 a**	**0.126 ± 0.010 b**	**0.154 ± 0.010 b**	**0.156 ± 0.012 b**	**0.137 ± 0.013 b**
Boron (B)	**0.597 ± 0.103 a**	**0.893 ± 0.059 b**	**0.757 ± 0.046 b**	**0.800 ± 0.050 b**	**0.894 ± 0.053 b**
Calcium (Ca)	73.2 ± 4.5 a	64.1 ± 3.6 a	65.0 ± 2.4 a	58.3 ± 3.0 a	58.2 ± 2.8 a
Copper (Cu)	0.378 ± 0.029 a	0.382 ± 0.021 a	0.410 ± 0.014 a	0.354 ± 0.016 a	0.365 ± 0.017 a
Iron (Fe)	2.00 ± 0.23 a	1.89 ± 0.13 a	1.87 ± 0.11 a	1.73 ± 0.08 a	1.96 ± 0.13 a
Magnesium (Mg)	107 ± 10 a	105 ± 7 a	118 ± 5 a	112 ± 4 a	117 ± 6 a
Manganese (Mn)	0.423 ± 0.054 a	0.506 ± 0.041 a	0.562 ± 0.031 a	0.507 ± 0.032 a	0.519 ± 0.026 a
Sodium (Na)	**1.18 ± 0.10 ab**	**1.26 ± 0.12 ab**	**1.02 ± 0.10 a**	**1.40 ± 0.09 b**	**1.30 ± 0.12 ab**
Sulphur (S)	132 ± 6 a	129 ± 4 a	127 ± 4 a	130 ± 3 a	131 ± 5 a
Zinc (Zn)	1.14 ± 0.09 a	0.91 ± 0.07 a	0.89 ± 0.04 a	0.93 ± 0.05 a	0.92 ± 0.05 a

Means ± SE within a row with bold font and different letters are significantly different (GLM, $p < 0.05$, n = 14 nuts for self-pollination and n = 37–62 nuts for other pollen parents).

Table S5. Total mineral nutrient contents per kernel (mg / kernel) in the mature kernels of macadamia cultivar '816' arising from different pollen parents

Nutrient	Maternal parent × pollen parent				
	'816' × '816'	'816' × '741'	'816' × '842'	'816' × 'A203'	'816' × 'A4'
Nitrogen (N)	**32.05 ± 2.54 ab**	**30.92 ± 0.98 a**	**37.62 ± 1.07 b**	**38.78 ± 1.04 bc**	**41.99 ± 1.38 c**
Phosphorus (P)	**4.65 ± 0.55 ab**	**4.58 ± 0.30 a**	**5.30 ± 0.25 ab**	**6.39 ± 0.33 c**	**6.33 ± 0.33 bc**
Potassium (K)	**6.43 ± 0.83 a**	**9.00 ± 0.66 a**	**9.79 ± 0.47 a**	**12.47 ± 0.67 b**	**11.50 ± 0.51 b**
Aluminium (Al)	**0.0057 ± 0.0013 a**	**0.0031 ± 0.0003 b**	**0.0042 ± 0.0003 b**	**0.0049 ± 0.0004 a**	**0.0043 ± 0.0004 ab**
Boron (B)	**0.013 ± 0.002 a**	**0.021 ± 0.001 ab**	**0.021 ± 0.001 ab**	**0.024 ± 0.001 bc**	**0.029 ± 0.002 c**
Calcium (Ca)	1.60 ± 0.14 a	1.53 ± 0.10 a	1.76 ± 0.08 a	1.75 ± 0.92 a	1.83 ± 0.08 a
Copper (Cu)	0.009 ± 0.001 a	0.009 ± 0.001 a	0.011 ± 0.001 a	0.011 ± 0.001 a	0.012 ± 0.001 a
Iron (Fe)	**0.042 ± 0.005 a**	**0.045 ± 0.004 a**	**0.051 ± 0.003 ab**	**0.053 ± 0.003 ab**	**0.064 ± 0.005 b**
Magnesium (Mg)	**2.40 ± 0.28 a**	**2.49 ± 0.18 a**	**3.21 ± 0.16 ab**	**3.44 ± 0.16 bc**	**3.58 ± 0.19 c**
Manganese (Mn)	**0.010 ± 0.001 a**	**0.012 ± 0.001 ab**	**0.016 ± 0.001 ab**	**0.015 ± 0.001 ab**	**0.017 ± 0.001 b**
Sodium (Na)	**0.027 ± 0.004 a**	**0.032 ± 0.004 ab**	**0.028 ± 0.003 a**	**0.043 ± 0.003 b**	**0.043 ± 0.004 b**
Sulphur (S)	**2.92 ± 0.26 a**	**3.09 ± 0.16 a**	**3.45 ± 0.12 a**	**3.93 ± 0.14 ab**	**4.16 ± 0.18 b**
Zinc (Zn)	**0.026 ± 0.003 ab**	**0.021 ± 0.002 a**	**0.024 ± 0.001 a**	**0.029 ± 0.002 b**	**0.029 ± 0.002 b**

Means ± SE within rows with bold font and different letters are significantly different (GLM, $p < 0.05$, n = 14 nuts for self-pollination and n = 37–62 nuts for other pollen parents).
